# 1,3-Dimethyl-1*H*-indole-2-carbonitrile

**DOI:** 10.1107/S1600536809024817

**Published:** 2009-07-04

**Authors:** Jiang-Sheng Li, Peng-Mian Huang

**Affiliations:** a School of Chemistry and Biological Engineering, Changsha University of Science & Technology, Changsha 410004, People’s Republic of China

## Abstract

The title compound, C_11_H_10_N_2_, crystallizes with two mol­ecules in the asymmetric unit, both of which are essentially planar (r.m.s. deviations = 0.014 and 0.016 Å). In the crystal, aromatic π–π stacking inter­actions occur [shortest centroid–centroid separation = 3.5569 (11) Å].

## Related literature

For the synthesis, see: Snyder & Eliel (1948 ▶).
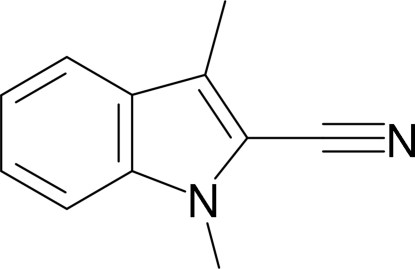


## Experimental

### 

#### Crystal data

C_11_H_10_N_2_
*M* *_r_* = 170.21
Monoclinic, 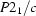

*a* = 8.8066 (18) Å
*b* = 15.359 (3) Å
*c* = 13.480 (3) Å
β = 95.67 (3)°
*V* = 1814.4 (7) Å^3^
*Z* = 8
Mo *K*α radiation
μ = 0.08 mm^−1^
*T* = 113 K
0.20 × 0.18 × 0.14 mm

#### Data collection

Rigaku Saturn CCD area-detector diffractometer
Absorption correction: multi-scan (*CrystalClear*; Rigaku/MSC, 2005 ▶) *T*_min_ = 0.985, *T*_max_ = 0.990
16175 measured reflections
4303 independent reflections
3462 reflections with *I* > 2σ(*I*)
*R*_int_ = 0.035

#### Refinement

*R*[*F* ^2^ > 2σ(*F* ^2^)] = 0.048
*wR*(*F* ^2^) = 0.132
*S* = 1.03
4303 reflections
238 parameters
H-atom parameters constrained
Δρ_max_ = 0.29 e Å^−3^
Δρ_min_ = −0.28 e Å^−3^

### 

Data collection: *CrystalClear* (Rigaku/MSC, 2005 ▶); cell refinement: *CrystalClear*; data reduction: *CrystalClear*; program(s) used to solve structure: *SHELXS97* (Sheldrick, 2008 ▶); program(s) used to refine structure: *SHELXL97* (Sheldrick, 2008 ▶); molecular graphics: *SHELXTL* (Sheldrick, 2008 ▶); software used to prepare material for publication: *CrystalStructure* (Rigaku/MSC, 2005 ▶).

## Supplementary Material

Crystal structure: contains datablocks global, I. DOI: 10.1107/S1600536809024817/hb5013sup1.cif

Structure factors: contains datablocks I. DOI: 10.1107/S1600536809024817/hb5013Isup2.hkl

Additional supplementary materials:  crystallographic information; 3D view; checkCIF report

## Figures and Tables

**Fig. 1.**
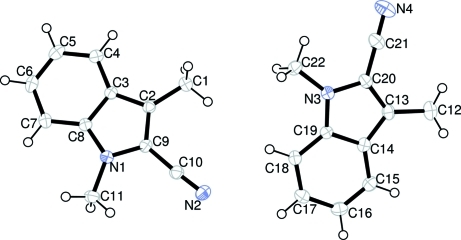
The two molecular structure of (I) in the asymmetrical unit with the atom-numbering scheme and 50% probability displacement ellipsoids.

**Fig. 2.**
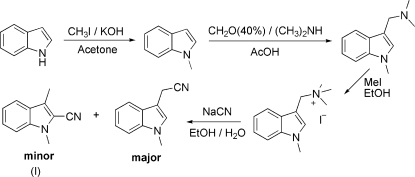
The formation of the title compound.

## supplementary crystallographic information

### Comment

The asymmetric unit of (I) comprises of two molecules (Fig. 1), in which the indole ring is each almost coplanar with a dihedral angle of 1.32 (7)° and 0.75 (7)°, respectively, between its pyrrole ring and fused benzene ring.

In the crystal packing, strong π-π stacking interactions help establishing the molecular packing.

### Experimental

The title compound was prepared according to the modified method of Snyder & Eliel (1948), as Scheme 1 shows. 1-Methyl-3-dimethylaminomethylindole was added to an ethanolic-aqueous solution (15%, 100 ml) of sodium cyanide (1.87 g, 0.038 mol), and then the resulting mixture was refluxed for 2 h, with the process monitored by TLC. After the reaction ceased, the reaction mixture was extracted with CH_2_Cl_2_ (3 × 50 ml), dried over anhydrous Na_2_SO_4_, and separated by flash chromatograhpy (ethyl acetate-petroleum 10/90 *v*/*v*) to provide the major product 1-methylindole-3-acetonitrile (yield 3.68 g,57%, m.p.328–330 K) and its isomeric substance 1,3-dimethyl-1*H*-indole-2-carbonitrile (yield 0.97 g,15%, m.p.339–340 K).  Colourless blocks of (I) were grown from a mixture of ethyl actate and petroleum ether (1:1 *v*/*v*).

### Refinement

All H atoms were positioned geometrically (C—H = 0.95–0.98 Å)and refined as riding with *U*_iso_(H) = 1.2*U*_eq_(CH) or 1.5*U*_eq_(CH_3_).

### Crystal data

**Table:**

C_11_H_10_N_2_	*F*(000) = 720
*M**_r_* = 170.21	*D*_x_ = 1.246 Mg m^−^^3^
Monoclinic, *P*2_1_/*c*	Mo *K*α radiation, λ = 0.71073 Å
Hall symbol: -P 2ybc	Cell parameters from 5208 reflections
*a* = 8.8066 (18) Å	θ = 2.0–27.9°
*b* = 15.359 (3) Å	µ = 0.08 mm^−^^1^
*c* = 13.480 (3) Å	*T* = 113 K
β = 95.67 (3)°	Block, colourless
*V* = 1814.4 (7) Å^3^	0.20 × 0.18 × 0.14 mm
*Z* = 8	

### Data collection

**Table:**

Rigaku Saturn CCD area-detector diffractometer	4303 independent reflections
Radiation source: rotating anode	3462 reflections with *I* > 2σ(*I*)
confocla	*R*_int_ = 0.035
Detector resolution: 7.31 pixels mm^-1^	θ_max_ = 27.9°, θ_min_ = 2.0°
ω and φ scans	*h* = −11→7
Absorption correction: multi-scan (*CrystalClear*; Rigaku/MSC, 2005)	*k* = −20→20
*T*_min_ = 0.985, *T*_max_ = 0.990	*l* = −17→17
16175 measured reflections	

### Refinement

**Table:**

Refinement on *F*^2^	Primary atom site location: structure-invariant direct methods
Least-squares matrix: full	Secondary atom site location: difference Fourier map
*R*[*F*^2^ > 2σ(*F*^2^)] = 0.048	Hydrogen site location: inferred from neighbouring sites
*wR*(*F*^2^) = 0.132	H-atom parameters constrained
*S* = 1.03	*w* = 1/[σ^2^(*F*_o_^2^) + (0.0783*P*)^2^ + 0.2443*P*] where *P* = (*F*_o_^2^ + 2*F*_c_^2^)/3
4303 reflections	(Δ/σ)_max_ = 0.003
238 parameters	Δρ_max_ = 0.29 e Å^−^^3^
0 restraints	Δρ_min_ = −0.28 e Å^−^^3^

### Special details

**Table:**

Geometry. All e.s.d.'s (except the e.s.d. in the dihedral angle between two l.s. planes) are estimated using the full covariance matrix. The cell e.s.d.'s are taken into account individually in the estimation of e.s.d.'s in distances, angles and torsion angles; correlations between e.s.d.'s in cell parameters are only used when they are defined by crystal symmetry. An approximate (isotropic) treatment of cell e.s.d.'s is used for estimating e.s.d.'s involving l.s. planes.
Refinement. Refinement of *F*^2^ against ALL reflections. The weighted *R*-factor *wR* and goodness of fit *S* are based on *F*^2^, conventional *R*-factors *R* are based on *F*, with *F* set to zero for negative *F*^2^. The threshold expression of *F*^2^ > σ(*F*^2^) is used only for calculating *R*-factors(gt) *etc*. and is not relevant to the choice of reflections for refinement. *R*-factors based on *F*^2^ are statistically about twice as large as those based on *F*, and *R*- factors based on ALL data will be even larger.

### Fractional atomic coordinates and isotropic or equivalent isotropic displacement parameters (Å^2^)

**Table:**

	*x*	*y*	*z*	*U*_iso_*/*U*_eq_	
N1	0.82816 (12)	0.53565 (7)	0.89373 (8)	0.0220 (2)	
N2	0.54466 (13)	0.37505 (8)	0.91305 (9)	0.0323 (3)	
N3	0.68373 (12)	0.02746 (7)	0.89897 (8)	0.0234 (3)	
N4	0.88484 (17)	−0.16898 (9)	0.90325 (11)	0.0447 (4)	
C1	0.94864 (16)	0.30650 (8)	0.85689 (11)	0.0294 (3)	
H1A	1.0143	0.2841	0.9142	0.044*	
H1B	0.9950	0.2936	0.7954	0.044*	
H1C	0.8482	0.2786	0.8544	0.044*	
C2	0.93090 (14)	0.40280 (8)	0.86704 (9)	0.0210 (3)	
C3	1.04387 (14)	0.46744 (8)	0.85879 (9)	0.0201 (3)	
C4	1.19845 (15)	0.46347 (8)	0.84089 (9)	0.0237 (3)	
H4	1.2462	0.4092	0.8308	0.028*	
C5	1.27868 (15)	0.54002 (9)	0.83840 (10)	0.0286 (3)	
H5	1.3834	0.5382	0.8271	0.034*	
C6	1.20941 (16)	0.62085 (9)	0.85218 (10)	0.0285 (3)	
H6	1.2680	0.6725	0.8488	0.034*	
C7	1.05797 (15)	0.62722 (8)	0.87060 (9)	0.0247 (3)	
H7	1.0114	0.6820	0.8800	0.030*	
C8	0.97653 (14)	0.54941 (8)	0.87480 (9)	0.0200 (3)	
C9	0.80186 (14)	0.44673 (8)	0.88817 (9)	0.0211 (3)	
C10	0.65833 (15)	0.40909 (8)	0.90293 (10)	0.0242 (3)	
C11	0.71576 (15)	0.60235 (9)	0.91016 (10)	0.0278 (3)	
H11A	0.7664	0.6514	0.9463	0.042*	
H11B	0.6382	0.5779	0.9495	0.042*	
H11C	0.6670	0.6226	0.8458	0.042*	
C12	0.43756 (19)	−0.16808 (9)	0.85253 (11)	0.0339 (3)	
H12A	0.4196	−0.1801	0.7825	0.051*	
H12B	0.3426	−0.1699	0.8818	0.051*	
H12C	0.5057	−0.2110	0.8837	0.051*	
C13	0.50716 (16)	−0.07991 (8)	0.86744 (9)	0.0239 (3)	
C14	0.43132 (15)	0.00185 (8)	0.86448 (9)	0.0223 (3)	
C15	0.27685 (15)	0.02613 (9)	0.84775 (9)	0.0258 (3)	
H15	0.1994	−0.0167	0.8352	0.031*	
C16	0.24020 (16)	0.11338 (9)	0.85001 (10)	0.0280 (3)	
H16	0.1363	0.1307	0.8392	0.034*	
C17	0.35452 (16)	0.17728 (9)	0.86808 (10)	0.0277 (3)	
H17	0.3258	0.2369	0.8689	0.033*	
C18	0.50620 (16)	0.15567 (8)	0.88453 (10)	0.0244 (3)	
H18	0.5827	0.1991	0.8961	0.029*	
C19	0.54362 (14)	0.06698 (8)	0.88352 (9)	0.0205 (3)	
C20	0.66012 (16)	−0.06165 (8)	0.88826 (9)	0.0241 (3)	
C21	0.78416 (17)	−0.12122 (9)	0.89733 (11)	0.0306 (3)	
C22	0.83057 (15)	0.07062 (9)	0.91704 (11)	0.0300 (3)	
H22A	0.8496	0.1053	0.8585	0.045*	
H22B	0.9112	0.0268	0.9295	0.045*	
H22C	0.8301	0.1088	0.9753	0.045*	

### Atomic displacement parameters (Å^2^)

**Table:**

	*U*^11^	*U*^22^	*U*^33^	*U*^12^	*U*^13^	*U*^23^
N1	0.0207 (5)	0.0209 (5)	0.0245 (6)	0.0012 (4)	0.0027 (4)	−0.0009 (4)
N2	0.0231 (6)	0.0319 (6)	0.0425 (7)	−0.0034 (5)	0.0063 (5)	−0.0043 (5)
N3	0.0229 (6)	0.0213 (5)	0.0262 (6)	0.0001 (4)	0.0027 (4)	0.0008 (4)
N4	0.0502 (9)	0.0360 (7)	0.0480 (9)	0.0171 (7)	0.0053 (7)	0.0000 (6)
C1	0.0278 (7)	0.0219 (6)	0.0387 (8)	0.0004 (5)	0.0039 (6)	0.0009 (5)
C2	0.0196 (6)	0.0219 (6)	0.0211 (6)	−0.0003 (5)	0.0001 (5)	0.0009 (5)
C3	0.0202 (6)	0.0211 (6)	0.0189 (6)	−0.0001 (5)	0.0007 (5)	0.0012 (4)
C4	0.0210 (6)	0.0268 (6)	0.0232 (6)	0.0013 (5)	0.0023 (5)	0.0017 (5)
C5	0.0211 (6)	0.0385 (8)	0.0264 (7)	−0.0046 (6)	0.0028 (5)	0.0034 (6)
C6	0.0296 (7)	0.0278 (7)	0.0277 (7)	−0.0104 (6)	0.0010 (6)	0.0027 (5)
C7	0.0283 (7)	0.0212 (6)	0.0242 (7)	−0.0019 (5)	0.0008 (5)	−0.0004 (5)
C8	0.0202 (6)	0.0220 (6)	0.0174 (6)	0.0005 (5)	0.0001 (5)	0.0007 (4)
C9	0.0199 (6)	0.0222 (6)	0.0210 (6)	−0.0018 (5)	0.0011 (5)	0.0010 (5)
C10	0.0226 (6)	0.0245 (6)	0.0254 (7)	0.0011 (5)	0.0022 (5)	−0.0022 (5)
C11	0.0246 (7)	0.0275 (6)	0.0321 (7)	0.0058 (5)	0.0064 (6)	−0.0014 (5)
C12	0.0435 (9)	0.0250 (7)	0.0330 (8)	−0.0067 (6)	0.0024 (6)	−0.0010 (5)
C13	0.0305 (7)	0.0211 (6)	0.0201 (6)	−0.0028 (5)	0.0028 (5)	0.0001 (5)
C14	0.0252 (7)	0.0236 (6)	0.0182 (6)	−0.0034 (5)	0.0030 (5)	−0.0010 (5)
C15	0.0237 (7)	0.0317 (7)	0.0220 (7)	−0.0034 (6)	0.0015 (5)	−0.0013 (5)
C16	0.0234 (7)	0.0355 (7)	0.0248 (7)	0.0037 (6)	0.0013 (5)	0.0005 (5)
C17	0.0300 (7)	0.0254 (6)	0.0276 (7)	0.0055 (6)	0.0024 (6)	0.0018 (5)
C18	0.0266 (7)	0.0213 (6)	0.0252 (7)	−0.0016 (5)	0.0019 (5)	0.0010 (5)
C19	0.0204 (6)	0.0220 (6)	0.0192 (6)	−0.0001 (5)	0.0018 (5)	0.0009 (4)
C20	0.0296 (7)	0.0200 (6)	0.0231 (6)	0.0026 (5)	0.0045 (5)	0.0013 (5)
C21	0.0382 (8)	0.0249 (6)	0.0292 (7)	0.0052 (6)	0.0053 (6)	−0.0001 (5)
C22	0.0216 (7)	0.0292 (7)	0.0384 (8)	−0.0025 (6)	−0.0013 (6)	0.0030 (6)

### Geometric parameters (Å, °)

**Table:**

N1—C8	1.3722 (16)	C9—C10	1.4217 (17)
N1—C9	1.3860 (16)	C11—H11A	0.9800
N1—C11	1.4566 (16)	C11—H11B	0.9800
N2—C10	1.1493 (17)	C11—H11C	0.9800
N3—C19	1.3724 (16)	C12—C13	1.4917 (18)
N3—C20	1.3897 (16)	C12—H12A	0.9600
N3—C22	1.4522 (17)	C12—H12B	0.9600
N4—C21	1.1474 (19)	C12—H12C	0.9600
C1—C2	1.4949 (17)	C13—C20	1.3774 (19)
C1—H1A	0.9800	C13—C14	1.4211 (18)
C1—H1B	0.9800	C14—C15	1.4071 (19)
C1—H1C	0.9800	C14—C19	1.4123 (18)
C2—C9	1.3754 (17)	C15—C16	1.3794 (19)
C2—C3	1.4177 (17)	C15—H15	0.9500
C3—C4	1.4072 (18)	C16—C17	1.410 (2)
C3—C8	1.4172 (17)	C16—H16	0.9500
C4—C5	1.3739 (19)	C17—C18	1.3730 (19)
C4—H4	0.9500	C17—H17	0.9500
C5—C6	1.404 (2)	C18—C19	1.4019 (17)
C5—H5	0.9500	C18—H18	0.9500
C6—C7	1.384 (2)	C20—C21	1.4209 (19)
C6—H6	0.9500	C22—H22A	0.9800
C7—C8	1.3978 (17)	C22—H22B	0.9800
C7—H7	0.9500	C22—H22C	0.9800
			
C8—N1—C9	107.39 (10)	N1—C11—H11C	109.5
C8—N1—C11	126.43 (11)	H11A—C11—H11C	109.5
C9—N1—C11	126.07 (11)	H11B—C11—H11C	109.5
C19—N3—C20	107.26 (11)	C13—C12—H12A	109.4
C19—N3—C22	126.58 (11)	C13—C12—H12B	109.5
C20—N3—C22	126.05 (11)	H12A—C12—H12B	109.5
C2—C1—H1A	109.5	C13—C12—H12C	109.5
C2—C1—H1B	109.5	H12A—C12—H12C	109.5
H1A—C1—H1B	109.5	H12B—C12—H12C	109.5
C2—C1—H1C	109.5	C20—C13—C14	105.86 (11)
H1A—C1—H1C	109.5	C20—C13—C12	126.36 (12)
H1B—C1—H1C	109.5	C14—C13—C12	127.78 (13)
C9—C2—C3	105.86 (11)	C15—C14—C19	119.34 (12)
C9—C2—C1	126.89 (12)	C15—C14—C13	133.08 (12)
C3—C2—C1	127.24 (11)	C19—C14—C13	107.58 (12)
C4—C3—C8	119.41 (11)	C16—C15—C14	118.59 (12)
C4—C3—C2	132.95 (11)	C16—C15—H15	120.7
C8—C3—C2	107.62 (11)	C14—C15—H15	120.7
C5—C4—C3	118.40 (12)	C15—C16—C17	121.03 (13)
C5—C4—H4	120.8	C15—C16—H16	119.5
C3—C4—H4	120.8	C17—C16—H16	119.5
C4—C5—C6	121.49 (13)	C18—C17—C16	121.76 (12)
C4—C5—H5	119.3	C18—C17—H17	119.1
C6—C5—H5	119.3	C16—C17—H17	119.1
C7—C6—C5	121.70 (12)	C17—C18—C19	117.34 (12)
C7—C6—H6	119.2	C17—C18—H18	121.3
C5—C6—H6	119.2	C19—C18—H18	121.3
C6—C7—C8	117.01 (12)	N3—C19—C18	129.57 (12)
C6—C7—H7	121.5	N3—C19—C14	108.50 (11)
C8—C7—H7	121.5	C18—C19—C14	121.94 (12)
N1—C8—C7	129.87 (11)	C13—C20—N3	110.79 (11)
N1—C8—C3	108.18 (10)	C13—C20—C21	127.91 (12)
C7—C8—C3	121.96 (12)	N3—C20—C21	121.29 (12)
C2—C9—N1	110.94 (11)	N4—C21—C20	178.97 (16)
C2—C9—C10	126.43 (12)	N3—C22—H22A	109.5
N1—C9—C10	122.63 (11)	N3—C22—H22B	109.5
N2—C10—C9	176.77 (14)	H22A—C22—H22B	109.5
N1—C11—H11A	109.5	N3—C22—H22C	109.5
N1—C11—H11B	109.5	H22A—C22—H22C	109.5
H11A—C11—H11B	109.5	H22B—C22—H22C	109.5
			
C9—C2—C3—C4	178.15 (14)	C20—C13—C14—C15	−179.38 (13)
C1—C2—C3—C4	−0.8 (2)	C12—C13—C14—C15	−0.1 (2)
C9—C2—C3—C8	−0.48 (14)	C20—C13—C14—C19	0.25 (14)
C1—C2—C3—C8	−179.42 (12)	C12—C13—C14—C19	179.58 (13)
C8—C3—C4—C5	−0.77 (19)	C19—C14—C15—C16	0.34 (18)
C2—C3—C4—C5	−179.27 (13)	C13—C14—C15—C16	179.94 (13)
C3—C4—C5—C6	−0.7 (2)	C14—C15—C16—C17	0.36 (19)
C4—C5—C6—C7	1.1 (2)	C15—C16—C17—C18	−0.3 (2)
C5—C6—C7—C8	−0.1 (2)	C16—C17—C18—C19	−0.5 (2)
C9—N1—C8—C7	179.28 (12)	C20—N3—C19—C18	−178.96 (13)
C11—N1—C8—C7	2.9 (2)	C22—N3—C19—C18	−2.4 (2)
C9—N1—C8—C3	−0.91 (14)	C20—N3—C19—C14	0.89 (14)
C11—N1—C8—C3	−177.24 (11)	C22—N3—C19—C14	177.41 (12)
C6—C7—C8—N1	178.39 (12)	C17—C18—C19—N3	−178.95 (12)
C6—C7—C8—C3	−1.40 (19)	C17—C18—C19—C14	1.23 (19)
C4—C3—C8—N1	−177.98 (11)	C15—C14—C19—N3	178.98 (11)
C2—C3—C8—N1	0.87 (14)	C13—C14—C19—N3	−0.71 (14)
C4—C3—C8—C7	1.85 (19)	C15—C14—C19—C18	−1.17 (19)
C2—C3—C8—C7	−179.30 (11)	C13—C14—C19—C18	179.14 (11)
C3—C2—C9—N1	−0.08 (14)	C14—C13—C20—N3	0.30 (14)
C1—C2—C9—N1	178.86 (12)	C12—C13—C20—N3	−179.04 (12)
C3—C2—C9—C10	179.90 (12)	C14—C13—C20—C21	−178.78 (13)
C1—C2—C9—C10	−1.2 (2)	C12—C13—C20—C21	1.9 (2)
C8—N1—C9—C2	0.62 (14)	C19—N3—C20—C13	−0.75 (14)
C11—N1—C9—C2	176.97 (12)	C22—N3—C20—C13	−177.29 (12)
C8—N1—C9—C10	−179.36 (11)	C19—N3—C20—C21	178.41 (12)
C11—N1—C9—C10	−3.01 (19)	C22—N3—C20—C21	1.86 (19)
C2—C9—C10—N2	−13 (3)	C13—C20—C21—N4	103 (10)
N1—C9—C10—N2	167 (3)	N3—C20—C21—N4	−76 (10)
